# Brain Organoids—A Bottom-Up Approach for Studying Human Neurodevelopment

**DOI:** 10.3390/bioengineering6010009

**Published:** 2019-01-18

**Authors:** Eyal Karzbrun, Orly Reiner

**Affiliations:** 1 Kavli Institute for Theoretical Physics and Department of Physics, University of California, Santa Barbara, CA 93106, USA; 2 Department of Molecular Genetics, The Weizmann Institute of Science, Rehovot 76100, Israel

**Keywords:** organoids, brain organoids, neurodevelopment, disorders

## Abstract

Brain organoids have recently emerged as a three-dimensional tissue culture platform to study the principles of neurodevelopment and morphogenesis. Importantly, brain organoids can be derived from human stem cells, and thus offer a model system for early human brain development and human specific disorders. However, there are still major differences between the in vitro systems and in vivo development. This is in part due to the challenge of engineering a suitable culture platform that will support proper development. In this review, we discuss the similarities and differences of human brain organoid systems in comparison to embryonic development. We then describe how organoids are used to model neurodevelopmental diseases. Finally, we describe challenges in organoid systems and how to approach these challenges using complementary bioengineering techniques.

## 1. Introduction

**Figure 1:**
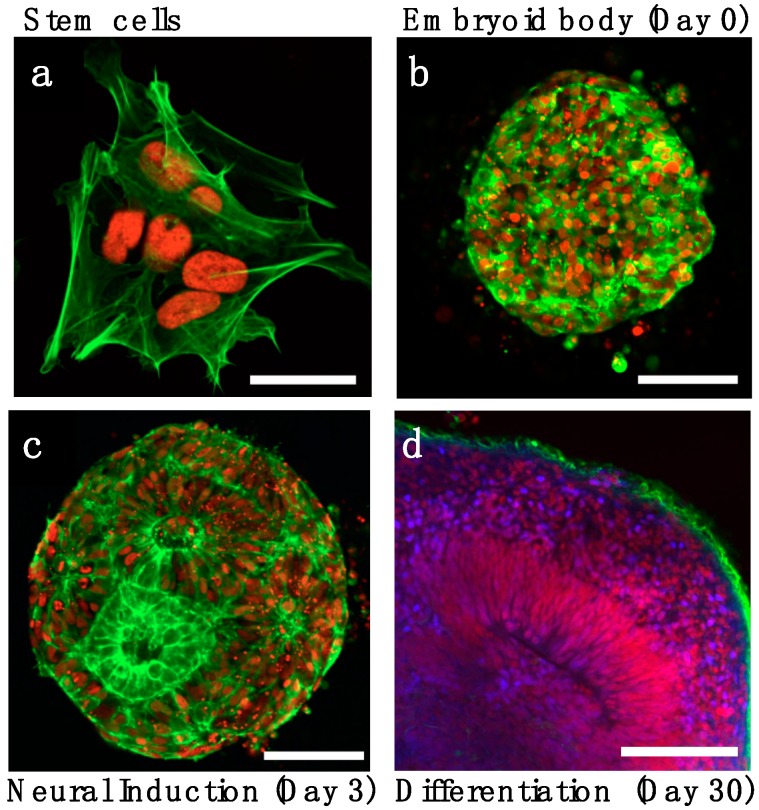
Stages of in vitro organoid development. (**a**) Stem cells with live reporters H2B-mCherry (red) and lifeact-GFP (green) are assembled into (**b**) 3D embryoid bodies. (**c**) Following neural induction, ventricle-like lumens appear within the 3D culture. (**d**) Neural differentiation and layer organization with neuronal progenitor (PAX6+, red) surrounding the lumen, an intermediate layer of sparse progenitors (DAPI, blue) and neuronal layer (TUJ1+, green) on the organoid periphery. Images adapted from Karzbrun et al. [7]. Scale bars are (**a**) 20 μm and (**b**–**d**) 100 μm.

Brain organoids are three-dimensional cell cultures that recapitulate key aspects of embryonic brain development [1,2,3,4,5,6] (Figure 1). Being derived from stem cells, they have the potential to recapitulate the fundamental aspects of neurodevelopment including neuronal differentiation, maturation and network formation. The release from the 2D dish and the transition to a 3D culture enables cells to move, deform, and collectively self-organize into structures that resemble the embryonic architecture. Ventricle formation, cortical layer organization, and neuronal migration are some of the organization features that are partially mimicked in brain organoids. Organoids can be considered a ‘blank slate’, as they lack regionally-defined guiding developmental signals. They thus offer the opportunity to quantitatively study the role of extrinsic biochemical and physical cues on the developmental process. In addition, the relative simplicity of brain organoids in comparison to in vivo animals, their accessibility and small size, and the high degree of experimental control turn them into a quantitative biology platform. These features make organoids an exciting system for a wide range of scientific disciplines. However, the limited and partial capacity of the in vitro development together with large batch-to-batch variability are among the shortcomings of the current organoid systems. Our hope is that these limitations can be overcome by new bioengineering techniques and approaches.

## 2. Recapitulation of In Vivo Neurodevelopment

In the following sections, we will compare the key steps in the in vitro development of brain organoids, and the corresponding in vivo events. We focus on four developmental stages: (I) Neuronal induction and fate patterning. (II) Cortical expansion and the subventricular zone. (III) Neurogenesis and cortical layer formation. (IV) Neuronal maturation and activity.

### 2.1. Neuronal Induction and Patterning

The embryonic development of the nervous system initiates with the derivation of the neural plate from the ectoderm, around post-conception week (PCW) 3. Neural fate is induced by the gastrula organizer (‘node’) through inhibition of BMP signaling from the surrounding ectoderm. BMP inhibition is mediated by a combination of signaling molecules including noggin and chordin. Patterning of the neural tissue into different regions, such as the brain and spinal cord, commences together with neural induction. For example, noggin, as mentioned above, participates in neural induction, and is also involved in regulation of dorsal-ventral patterning [8]. An additional important morphogen is sonic hedgehog (SHH), which is secreted by the notochord, and participates in patterning of the dorsal ventral axis, where high levels of SHH lead to ventral fates. 

How does neural induction and patterning occur in organoids? The organoid protocols begin with the formation of three-dimensional cell cultures (embryoid bodies) which float in nonadherent culture wells and contain thousands of cells (Figure 1). Embryoid bodies are cultured in a stem cell media containing growth human basic fibroblast growth factor (BFGF) and knockout serum replacement. After several days of culture, BFGF is withdrawn and neuronal induction media is applied for 7–12 days. Currently, two main approaches have been used for neuronal induction. One approach relies on specific molecules, such as SMAD and WNT inhibitors, to specify neuronal fate and to exclude differentiation into mesoderm and ectoderm lineage [9,10,11]. This approach attempts to mimic the signaling molecules secreted by the organizer, and is similar to two-dimensional neuronal induction protocols [12,13]. The second approach utilizes the notion that differentiation into neuronal fate occurs by default in the absence of external factors [14,15,16]. We begin by describing the first approach.

For induction of forebrain neuronal progenitors (FOXG1+ and/or OTX1/2+), a cocktail of TGFβ, BMP, and WNT inhibitors is applied for a week [10,11,17,18,19,20]. An alternative patterning approach is the application of an SHH inhibitor [21]. By the end of the first week of induction, cells in the embryoid body attain an anterior dorsal fate (PAX6+ and EMX1/2+). To attain a ventral anterior fate patterning (NKX2.1+) a second period of induction with SHH activation and WNT inhibition is required [21,22,23,24]. Similar approaches, using different signaling molecules, were developed for optic cup [25,26,27] and spinal cord [28] organoids from human cells and inner ear organoids from mouse cells [29]. 

Neural induction can also be achieved in the absence of patterning molecules [14,15,16]. In vitro there is no BMP secretion from the ectoderm, and thus, in principle, BMP inhibition is not required. Self-patterning protocols being with an induction phase in a defined serum-free media including insulin to support cell survival. After several days to a week, a neuroepithelium appears at the surface of the embryoid body. At this point, the embryoid body is embedded in Matrigel, which forms an extracellular matrix (ECM) support for the developing organoid. ECM has been shown to be important for correct polarization of neuronal progenitors in vitro [30]. The long-term culture of organoids can be carried out in spinning bioreactors to improve growth conditions [14]. In the absence of patterning molecules, organoids exhibit a rich diversity of cellular identities, including dorsal forebrain (FOXG1+), ventral forebrain (OTX2+, GAD1/2+), hindbrain (KROX20+, ISL1+), hippocampus (NR2+), hem (WNT2B+), choroid plexus (TTR+), and retina (CRX+) [14,15,16]. 

To summarize, neuronal patterning using small molecules has been successful in establishing a wide range of neuronal fates, but at the price of forming region-specific organoids. This is in contrast to the embryonic brain, where multiple regions and cellular fates coexist and simultaneously develop. Avoiding molecular patterning allows researchers to attain whole-brain organoids, with multiple cellular identities in a single organoid, at the price of increased organoid-to-organoid variability and the appearance and inclusion of non-neuronal fates. 

A future challenge is to accomplish reproducible whole-brain organoids, with controllable progenitor populations. One possible solution is to apply spatiotemporal combinations of patterning signals, such that different regions of the organoid will be exposed to a different mixture of developmental signals. For example, opposing concentration gradients of SHH and BMP signaling molecules were previously used to generate a mixed population of neuronal subtypes in 2D culture [31]. The gradients were created in a microfluidic device, and exhibited almost linear profiles over a millimeter sized reaction chamber. It would be interesting to apply such molecular gradients on a developing organoid, and observe whether this would control the progenitor populations and their position in the organoids. For this, one must match the length scale of morphogen with the organoid diameter. An alternative approach is optogenetics. Optogenetic tools can be used to induce activation or overexpression of morphogenetic signals, similar to recent demonstrations of Rho [32] and Bicoid [33] activation during early Drosophila embryogenesis. Optogenetics can generate very local signals and complex patterns over time, while microfluidics will mostly generate continuous gradients. However, small molecule signaling, which are used in microfluidics setups, may offer a richer repertoire than optogenetics. Finally, 3D bioprinting of may serve as another solution. In this scenario, prepatterned progenitors will be assembled into a single 3D structure. These methods are further discussed in the last section. 

### 2.2. Cortical Expansion and the Subventricular Zone

The evolutionary expansion of the neocortex is believed to contribute to the higher cognitive capacity of humans [34,35,36]. The human neocortex holds about 16 billion neurons, whereas our closet relative the chimpanzee brain hosts six billion neurons [37,38]. It is believed that this expansion is largely due a subpopulation of neuronal progenitors which are dramatically expanded in humans. An exciting research direction is the study of evolutionary driven cortical expansion in brain organoids [39,40,41,42,43,44,45]. The in vitro platform allows researchers to compare the developmental dynamics of human, apes, and mice derived organoids under similar experimental conditions. In this section we describe recent advances in this field. We begin with a brief description of cortical proliferation and neurogenesis.

In the neocortex, neurogenesis occurs at two major proliferative zones. The ventricular zone (VZ) is located at the apical side of the cortex, adjacent to the brain ventricle, and is the main proliferative zone for non-primate animals. A major progenitor population in the VZ is radial glia, bipolar-shaped cells that span the entire cortex and express unique markers including PAX6 and SOX2. The second proliferative zone is the subventricular zone (SVZ), which contains basal progenitors [46]. In primates, the SVZ can be further distinguished into outer and inner SVZ (oSVZ, iSVZ), separated by an inner fiber layer. In fetal human brains, the oSVZ is several folds larger in size in comparison with the VZ, suggesting it is a major source of neuron production during human neurodevelopment [35,47,48,49,50,51,52,53]. During neurogenesis, the pool of progenitor cells is depleted, while the population of neurons grows. The rates of proliferation and neurogenesis change during development, and their balance is thought to determine the species dependent brain size.

Brain organoids successfully mimic several features of cortical development [7,11,14,15]. Organoids contain a ventricle-like lumen, surrounded by a proliferative VZ-like region, approximately 100 micron thick. The apical progenitors exhibit the morphology of radial glia with thin apical and basal processes, and express radial glia marker genes (PAX6, SOX2, BRN2, NESTIN, and SOX1/2) [11,14,15]. The progenitors exhibit apical-to-basal interkinetic nuclear motion that is coupled with cell cycle, and is a hallmark of radial glia cells. Single cell RNA sequencing and immunostaining revealed that at day 30, the identity of the majority of the organoid cells relates to apical progenitors (PAX6+TBR2−). Later on (days 50–60), there is an increase in the population of basal progenitors (TBR2+), and a decrease in the population of apical progenitors. Furthermore, the TBR2+ basal progenitors are localized to a SVZ-like layer, which is spatially distinct from the VZ. Notably, oSVZ progenitors were found in large numbers in day 84 organoids [11]. The oSVZ progenitors expressed unique markers such as HOPX, FAM107A, and PTPRZ1 [53,54]. The late expression of oSVZ-exclusive markers in the organoid is consistent with their appearance during gestational weeks 15–20 [54]. Importantly, oSVZ-like cells are produced in human derived organoids, but do not appear in mouse derived systems [9,11,14,45]. Studies revealed additional interspecies differences in the neuronal progenitor population derived from humans, apes, and mice. These include a higher proportion of basal progenitors in human organoids [42], elongation of the times for prometaphase and metaphase of human neuronal progenitors [42], longer proliferative periods of human neuronal progenitors which overlap with the neurogenesis period [44], and slower neuronal maturation dynamics [44,45]. Overall, brain organoids successfully reveal human specific dynamics of neuronal progenitors. However, so far, the dramatic expansion of the oSVZ has not been observed in vitro. 

Cortical expansion is also associated with gyrification [36,55,56]. Large brain species, such as primates, exhibit a folded cortex, whereas as small brain species, such as mice, exhibit a smooth cortex. Thus, it seems that gyrification has evolved as a way of packing a large surface cortex into a small volume skull. However, the physiological mechanism behind gyrification is not fully understood. A possible explanation for folding is differential growth. As the outer regions of the brain grow faster than the interior, strain accumulates, leading to a mechanical instability which results in wrinkling. This phenomenon has been observed in mathematical models, computer simulations, and swelling gels models [57,58,59,60]. Recently, we have observed folding of human brain organoids cultured in confined microdevices [7,61]. Despite the early onset of gyrification in the organoids, our results indicate that the organoid surface folding is driven by a combination of differential growth and tension at the apical surface. Furthermore, we observed a periodic folding pattern with a peak-to-peak distance that scales with tissue thickness, consistent with theoretical models of differential growth, and an indication that the folding pattern is controlled by tissue mechanics. Interestingly, we have observed that both cell mechanics and organoid folding are abnormal in *LIS1+/−* mutant cell lines. A previous study showed that PTEN deletion stimulates proliferation and increases organoid folding [62]. The implications of genetic mutations on organoid and cortical folding are discussed later in the review.

One of the main challenges of organoid systems is the limited growth during the in vitro culture compared to the in vivo expansion. While embryoid bodies may initially exhibit exponential growth, this growth rate reduces over time. The reduced growth rate may be due to the biochemical composition of the media, as well as inefficient transport of nutrients into the thick 3D structure. In the developing embryo, vasculature intercalates into the brain and supplies nutrients and oxygen. The vasculature is differentiated from the endoderm germ layer, which is absent in brain organoids. 

In the bioengineering section, we discuss how embedding a vasculature into organoids may improve culture conditions. Increase in growth rate, may lead to significant expansion of oSVZ population, which is crucial for recapitulating in vivo development. 

### 2.3. Neurogenesis and Cortical Layers Formation

The neocortex is composed of six horizontal neuronal layers, which are essential for proper brain function. Each layer composes a different class of neurons, which are interconnected in a stereotypic fashion. For example, cells in layers V and VI often project out of the cortex, and cells in layer II form connections within the cortex. The spatial layer organization arises from temporally ordered generation and radial migration of neurons, from the ventricular and subventricular zones. Interestingly, the temporal order of neurogenesis is persevered during in vitro differentiation both in 2D and 3D cultures [44,45]. In vitro, Layer IV neurons (CTIP2+) are generated at day 20 following neuronal induction, and upper layer II–IV neurons (SATB2+, RORB+, KCNIP2+, and MDGA1+) appear at day 70. These time scales are consistent with the 45-day interval between layers IV and VI in the human embryo [41]. In organoids, deep layer neurogenesis peaks at days 52–76, whereas the neurogenesis of superficial layers peaks at days 76–136 [10]. Layer II/III neurons (CUX1+ and BRN2+) were reported to appear around day 80 [11]. The appearance of astrocytes occurs during later stages of cortical development. In more mature organoids (day 180), 20% of the cells expressed GFAP and exhibited astrocytes morphology and contained numerous glycogen granules [10]. Overall, neuronal and SVZ layer expansion are observed from days 56 to 84, at the expense of VZ reduction. 

To better understand the developmental timeline in 3D cultures, bulk RNA-sequencing from organoids was compared to transcriptional profiles of the post-mortem embryonic human brain transcriptomes data bases [63,64,65]. In one study, gene expression patterns in organoids at day 52 and 74 correlated best with post-conception weeks (PCW) 19–24 [10]. In another study, day 31 organoids displayed maximum correlation with PCW 9 [66]. In yet another study, day 26–54 organoids were related to 8–9 PCW, whereas day 100 organoids were more related to 17–24 PCW [11]. Taken together, these data indicate that organoids are capable of modeling early human neocortical development. 

We next describe the spatial organization of cortical neurons in 3D cultures. Organoids at day 30 of differentiation contain a ventricle-like lumen surrounded by a layer of apical progenitors (PAX6+), which is covered by an additional layer of early-born layer V neurons (CTIP2+) and an external layer of type VI neurons (TBR1+), suggesting initiation of cortical plate layer formation [11,14]. Around day 80 or later, the neuronal layer exhibit distinct separation into an early-born layer V (CTIP2+) and a late-born superficial layer III/IV (SATB2+ and BRN2+) [10,11,14]. Interestingly, layer I REELIN+ cells typically appear as an upper layer in organoids, similar to their position in vivo. In the cortex, Cajal–Retzius cells emerge from three different sources and typically from the borders of the developing cortex including the cortical hem. However, it is yet not clear what is the identity of the progenitors of these cells in vitro. 

Overall, brain organoids display limited spatial distinction between neuronal layers. The interlayer mixing can arise from an overlap in the temporal dynamics of neurogenesis, as well as from issues in migration. These discrepancies may stem from the difference in boundary conditions between the developing brain and organoid. The brain is enveloped and sealed by the meninges, whereas the organoid surface is exposed to the media. Meninges secrete substances such as retinoic acid that affect neuronal development by communicating with the adjacent radial glial endfeet [67]. In addition, molecules and morphogens that are secreted by the organoid and are essential for development may diffuse out into the culture. For example, REELIN is a glycoprotein which is secreted by layer I neurons, and is essential for neuronal migration and positioning. In the brain, REELIN secretion establishes a concentration gradient that is maximal at the basal surface, and minimal at the apical surface. This gradient guides neurons through their apical to basal migration. Thus, it is possible that the open boundary conditions of the organoid basal surface interfere with the formation of a REELIN concentration gradient, which could explain the limited neuronal lamination. This hypothesis could be tested by engineering an elastic membrane that would coat the organoid, and provide boundary conditions similar to the developing brain. Such a membrane should be permeable to water, salts and small nutrients but impermeable to proteins. Furthermore, it should be sufficiently elastic to stretch around the growing organoid, without causing significant mechanical stress. Such a membrane coating could also be used to study whether mechanical tension affects the developing organoid.

The cortex is composed of 80% excitatory glutamatergic neurons which are generated from ventricular and subventricular zones. Inhibitory GABAergic neurons constitute the remaining 20% of neurons in the cortex and mostly originate from the ganglionic eminences of the brain [68,69]. These neurons migrate to the cortex, and integrate into cortical networks. Ventrally patterned organoids enable researchers to study the neurogenesis and migration of GABAergic neurons [11,21,23,24,70]. In midbrain organoids, dopaminergic (DA) (TH+) neurons appear at day 38 [11]. By day 56, TH+ neurons express the floor-plate marker FOXA2, and dopamine transporter (DAT), as well as other DA markers (NURR1 and PITX3). In day 40 hypothalamic organoids, peptidergic neuronal markers, including POMC, VIP, OXT, and NPY, were observed [11]. At day 105, single cell RNAseq of subpalium organoids revealed several populations including ventral neural progenitors, GABAergic neurons (*DLX1*, *GAD1*, *SCG2*, and *SST)*, oligodendrocyte progenitors (*OLIG2* and *SOX10*), and astroglia (*TTR* and *SLC13A4*) [23].

The integration of inhibitory neurons into cortical organoids was studied by physically coupling ventral and dorsal prepatterned organoids [21,23,24]. Researchers observed a unidirectional cell migration from the ventral to dorsal organoid. The majority of migrating cells was post-mitotic and expressed GABAergic (GAD1+) markers. Region specific markers were also observed including MGE (SOX6+), LGE (COUP-TFII+), and CGE (SP+) [21]. In another study, migrating cells expressed ventral markers (*DLX1*/2/5/6) as well as cortical interneuron markers (*GAD1/2*, *VGAT*, and *CELF4*) [23]. Migration was initially observed at day 30, and the density of migrating cells peaked at day 46 and continued at list until day 80 [21]. Cells migrated in a saltatory pattern with single or branched leading process, which is similar to the migration pattern observed in rodents [21,23,24]. Notably, the motion was disturbed in the presence of CXCR4 receptor antagonist, a receptor that plays a role in interneuron migration [23], and in the presence of blebbistatin, a myosin II inhibitor [24]. In addition, interneurons which migrated into the dorsal organoid exhibited changes in gene expression profiles, displayed increased branching complexity, and seemed to form microcircuits with glutamatergic neurons [23]. 

### 2.4. Neuronal Maturation and Activity

Multiple studies indicate that organoid derived neurons are functionally mature, display spontaneous firing activity, active synaptic connections, and long-range neuronal response [10,11,14,16,23,24,71]. The neurons mature over several months of culture, exhibiting changes in firing properties, and the appearance of synaptic markers. GFP-labeled axon projections display complex branching and growth cone behavior and project long-range axons in a manner reminiscent of axon bundling. In addition, GFP+ neurons exhibited complex neuronal morphology with spine-like structures in close association with presynaptic SV2+ puncta [11]. Organoids showed colocalization of presynaptic protein synapsin-1 and postsynaptic protein PSD-95 puncta, and exhibited long range neuronal response to local electric stimulation or current injection [10]. Puncta of VGAT and VGLUT1 also suggested that both glutamatergic and GABAergic synapses were present [16]. Astrocyte maturation was study in cortical organoids cultured for 20 months [19]. Over this period, astrocytes developed a characteristic star-shaped morphology with increasing branching number over time. Finally, addition of platelet-derived growth factor AA and insulin-like growth factor 1 leads to generation of oligodendrocytes (PLP1+ and MYRF+) in cortical organoids [72]. Remarkably, their maturation, at around week 20 in culture, results in myelination of axons as observed by immunostaining and electron microcopy. 

So far, neuronal activity measurements in organoids have been mainly short-term. However, the in vitro culture offers a great opportunity to carry out studies of neuronal activity, over developmental time scales, which are impossible in vivo. Thus, it would be interesting to couple brain organoid culture with long term electrical recording, and possibly a closed-loop feedback [73]. Continuous electric stimulation into the organoid can be used to emulate input from other brain regions, and study its effect on neuronal maturation and network formation. This approach has been previously successful in 2D cortical cultures, where a closed-loop feedback was used to control bursting activity [74]. Using a similar approach in developing organoids will enable us to study how stimulation guides cortical network formation in vitro. 

## 3. Organoids for Neurodevelopmental Disease Modeling

**Figure 2:**
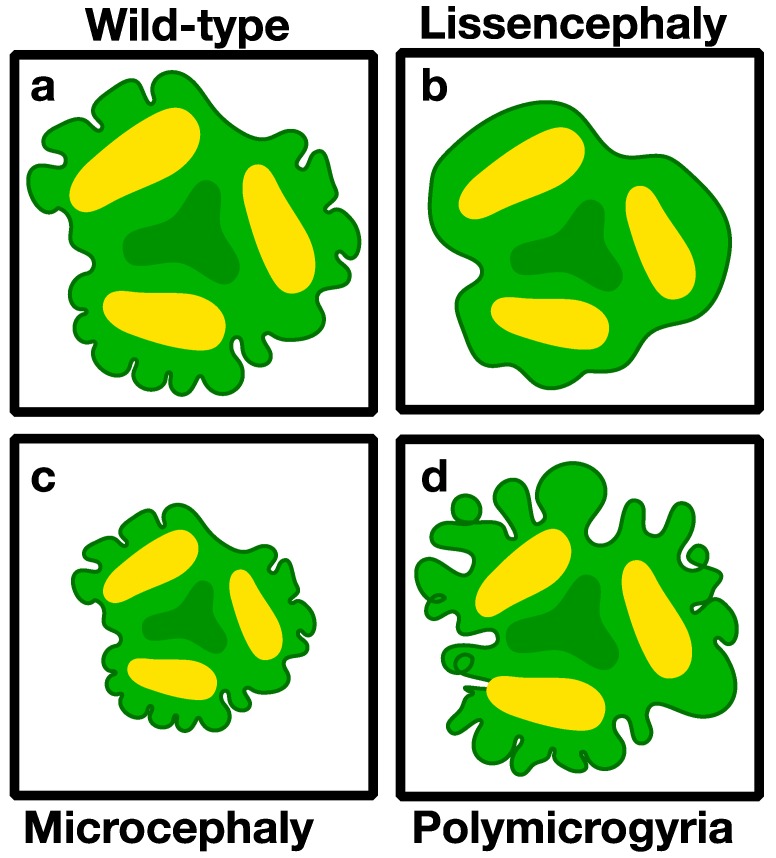
Schematic presentation of brain malformations that can be modeled in brain organoids. (**a**) Normal brain organoids (**b**) lissencephaly (smooth brain) (**c**) microcephaly (small brain), and (**d**) polymicrogyria (extra brain folds).

The cerebral cortex is a mammalian-specific brain region that is the center of higher order cognitive functioning, and the site of origin of many devastating neurodevelopmental, neuropsychiatric, and neurological diseases. Ethical and practical concerns limit access to human embryos, leading researchers to rely heavily on model systems, such as the mouse, as a surrogate to understand human brain development and disease. However, mouse models often cannot uncover disease etiology and may not accurately presage drug efficacy as there are key differences between the mouse and human cerebral cortex. Until recently, these limitations have been insurmountable as human tissues were largely inaccessible. Technical innovations have now allowed for the generation of brain organoids from human pluripotent stem cells (reviews [4,40,75,76,77,78]). These organoids recapitulate many aspects of human brain development, are amenable to gene editing, and have revolutionized our ability to model disease pathophysiology (Figure 2). Notably, these advances were preceded and greatly aided by parallel innovations in stem cell technology, reprogramming abilities [79], and improvements in genome-editing technology, especially CRISPR/Cas9 [80,81,82]. 

### 3.1. Genome Engineering of Stem Cells for Organoids

The possibilities of genome editing opened numerous applications for organoid studies. We will discuss here two possibilities, the first will related to disease modeling and the second will relate to fluorescent reporters. In the case of disease models, gene editing allows us to introduce a battery of different allelic mutations in a single gene using the same human ES or iPSC. Alternatively, if the mutant cells are patient-derived, it is possible to precisely correct the mutation. This approach is of enormous practical significance since each individual has a different genetic background and at the present time it is not yet possible to interpret the functional implications of all the single bases changes that can be identified in one person. These genetic variations have a huge impact on gene expression, resulting in difficulties to interpret RNA-seq experiments. The uniformity of the genetic background in this case is even higher than that observed between different individual mice on an inbred background. The technology and precision of genome editing is advancing rapidly. The major drawbacks include inefficiency of precise base editing and off-target activities. Several approaches were used to reduce these drawbacks and include the use of mutant Cas9 enzymes that cannot introduce a double strand break. These include Cas9^D10A^, also known as nickase [82], but also the development of ’base editing’, a new approach to genome editing that enables the direct, irreversible conversion of one target DNA base into another in a programmable manner, without requiring dsDNA backbone cleavage or a donor template [83]. Knock-in or transgenesis approaches allow us to introduce fluorescent tags. Fluorescent reporters can be used for labeling of specific cell types using a knock-in to a gene that is cell type restricted, for example, knock-in of the *Foxg1* locus in mouse embryonic stem cells allows for distinguishing between the medial pallium and the hem [84]. To screen for conditions to enrich for medial ganglionic eminence (MGE)-like organoids, a NKX2-1-GFP reporter hESC line was used [24]. Alternatively, fluorescently-tagged cells were used to label organoids grown using different conditions [21], where organoids grown under conditions to induce ventral identity were labeled with GFP and those grown under conditions to induce dorsal identity were labeled with tdTomato. Another type of cell-specific reporter was used to indicate in which cells a somatic mutation occurred using local induction of Cre-recombinase [85]. Fluorescent reporters labeling cell type specific organelles were generated for the community by the Allen brain institute and currently include 28 cellular structures (https://www.allencell.org/cell-catalog.html). These cell lines can be used for generation of human brain organoids.

### 3.2. Modeling Genetic Diseases Associated with Brain Structure

#### 3.2.1. Microcephaly (Small Brains)

Microcephaly was the first genetic developmental brain disease to be modeled in brain organoids (Figure 2a,c) [14]. Microcephaly means a small brain, and it can be detected at birth or develop later during early childhood. This disease can arise from multiple conditions, some of which are genetic and may be due to problems existing already in the neuronal stem cells. In that study [14], iPSCs were reprogrammed from a patient with compound heterozygous truncating mutations in *CDK5RAP2*, a known primary microcephaly gene [86,87,88]. Most strikingly, the mutated cerebral organoids were smaller. The cell biology of the progenitors was affected, their numbers were reduced, and they demonstrated distorted spindle orientation and premature neuronal differentiation [14]. The specificity of the mutation was verified by a rescue experiment in which reintroduction of the CDK5RAP2 protein reduced the phenotype. Furthermore, knockdown of *CDK5RAP2* in control cells recapitulated the main features of the patient derived mutant cells [14]. The observed phenotype was attributed to malfunctioning of the centrosome. Mutations in centrosomal proteins, including CDK5RAP2, cause primary microcephaly and Seckel syndrome [87,89,90,91,92,93,94,95,96,97,98,99]. 

The cell biology of progenitors is an important feature in the developing brain. Abnormal mitotic spindle function can also result in a reduction of brain size. Mutations in the *ASPM* gene (abnormal spindle-like microcephaly-associated) are associated with microcephaly primary type 5 (MCPH5). These mutations are the most common cause for microcephaly, leading to reduced brain size, congenital failure and intellectual disability [86,100,101]. Organoids developed from a patient with mutations in the *ASPM* gene were smaller, and both the progenitors in the ventricular zone and the outer subventricular zone were reduced [102]. The structure of the spindle is determined also by proteins that regulate microtubules. Therefore, it is not surprising that mutations in KATNB1, encoding the p80 subunit of the microtubule-severing enzyme katanin, cause severe microcephaly with simplification of cortical gyri and sulci or microlissencephaly [103]. Brain organoids derived from a patient-derived iPSCs, contained less neurons which failed to migrate out [104]. 

#### 3.2.2. Macrocephaly (Large Brains)

Increased brain size is also associated with brain developmental diseases. One of the underlying causes may be due to increased neuronal stem cell proliferation, which is regulated by several key signaling pathways. The Notch pathway is known to play an evolutionary conserved role in regulation of neurogenesis [47,105,106]. Recent studies demonstrated the role of human-specific paralogs of the NOTCH2 gene that regulate neural progenitor proliferation and neuronal differentiation [107,108]. Interestingly, these genes are located in a deletion/duplication syndrome, associated with macrocephaly (large brain), microcephaly (small brain), autism, and schizophrenia [107,108,109]. Duplications of *NOTCH2NL* are present in patients affected by macrocephaly, whereas microcephaly is seen in patients with *NOTCH2NL* deletions. Furthermore, NOTCH2NL expression downregulated neuronal differentiation genes and delayed differentiation in both mouse and human cortical organoids [107]. In a similar way, it was shown that NOTCH2NL increases neural progenitor self-renewal through symmetric proliferative divisions in human embryonic stem cells [108]. It has been suggested that the physical interaction between NOTCH2NL with NOTCH2 may activate NOTCH2 in a non-cell-autonomous manner [107]. Alternatively, or in addition to, it has been proposed that NOTCH2NL blocks the expression of DLL1 Notch receptors, leading to promotion of neuronal differentiation [108]. 

The mammalian target of rapamycin (mTOR) pathway is known to control growth and metabolism by activating anabolic processes and suppressing catabolic processes [110]. Abnormalities in the mTOR pathway result in a significant number of brain disorders, such as pediatric brain tumors, autism, seizure, learning disability, and mental retardation [111]. Mutations in genes associated with the mTOR pathway result in macrocephaly [112,113,114,115,116]. Cerebral organoids generated from cells with a mutation in one of the mTOR pathway genes exhibited significantly larger and substantially folded structures [62]. The deletion of *PTEN*, which results in the activation of the mTOR pathway, allowed for sustained cell cycle re-entry, expansion of the progenitor population, and delayed neuronal differentiation. Therefore, the increased tissue size resulted from multiple changes 

#### 3.2.3. Lissencephaly (Smooth Brain)

Lissencephaly means a smooth brain (Figure 2a,b). Yet, upon further characterization it is clear that there are additional problems in cortical organization, which are due to abnormal neuronal migration. Children born with this condition do not research measurable developmental stages and most of them have epilepsy. Whereas, neuronal migration features of the disease were successfully modeled in mouse models, the smooth brain phenotype cannot be recapitulated due to the smoothness of the mouse brain. *LIS1* was the first gene identified associated with a neuronal migration disorder [117]. Genomic deletions that encompass more than the *LIS1* gene, result in a more severe outcome and is known as Miller–Dieker syndrome (MDS) [118]. Three studies modeled *LIS1*-related lissencephaly [7,119,120]. Two of the studies used iPSCs from MDS patients [119,120], while the third used hESC in which the *LIS1* gene was edited using CRISPR/Cas9 [7].

In two of the studies, the sizes of the organoids derived from patient iPSCs were significantly smaller than the control ones [119,120]. There were several differences which could account for the decreased size. The ratio of symmetrical divisions versus asymmetrical ones differed from the control, thus resulting in premature differentiation [119,120]. Increased apoptosis rates were also noted in one of the studies [119]. The patient derived organoids exhibited a small reduction in RG progenitor cells with a complementary increase in CTIP2 positive neurons. Furthermore, there was a clear reduction in oRG as has been evident from single-cell RNA-seq. The MDS oRG cells were also imaged and they exhibited longer mitosis periods. The second study suggested an effect on microtubule stability since they revealed decreased microtubule acetylation [120]. They also detected abnormalities in the structure of the adherens junctions at the ventricular zone which has been postulated to reduce Wnt-signaling. Part of the phenotypic alterations was rescued by Wnt activation. The third study used a unique on-chip organoid model, where the growth of the organoids is limited in the z-axis, resulting in a quasi-3D structure [7]. The engineered device enabled long-term live cell imaging throughout growth, sustained more uniform development of the organoids, and the cell-death that is usually observed in core of the organoid was eliminated. In this set-up the growing organoids developed folds, which continued to develop over time. These folds served as a model for brain folding. The *LIS1*-mutant organoids exhibited reduced and more variable folds, with a longer peak-to-peak distance between folds; however, the height of the folds did not differ from the wild type. At the cell biological level, a significant reduction was noted in the speed of nuclear motion towards the ventricle, which could be attributed to cytoplasmic dynein activity. In addition, most of the wild type nuclei spent most of their time in the outer part of the growing organoid, and have undergone expansion due to their time in S-phase, resulting in differential expansion of the organoid. In contrast, the nuclei in *LIS1* mutant cells did not obey this rule, resulting in reduced differential expansion, consistent with the reduction in folding. Comparison of gene expression between the WT and *LIS1* mutant on-chip organoids revealed a significant difference in the gene expression module related to the extracellular matrix (ECM), which is difficult to associate with known LIS1 functions. This finding may be of interest in view of the observed folding in these on-chip organoids. Of note, the ECM has been suggested to play an important role in the evolutionary expansion of the neocortex and its gyrification, especially within a unique subpopulation of basal or outer radial glia progenitors [15,43,121]. Thus, it may be an interesting model to help understand additional abnormalities of brain folds such as polymicrogyria (extra brain folds) (Figure 2a,d) and evolutionary processes involved in the emergence of brain folds. 

## 4. Bioengineering Challenges and Opportunities in Brain Organoids

**Figure 3:**
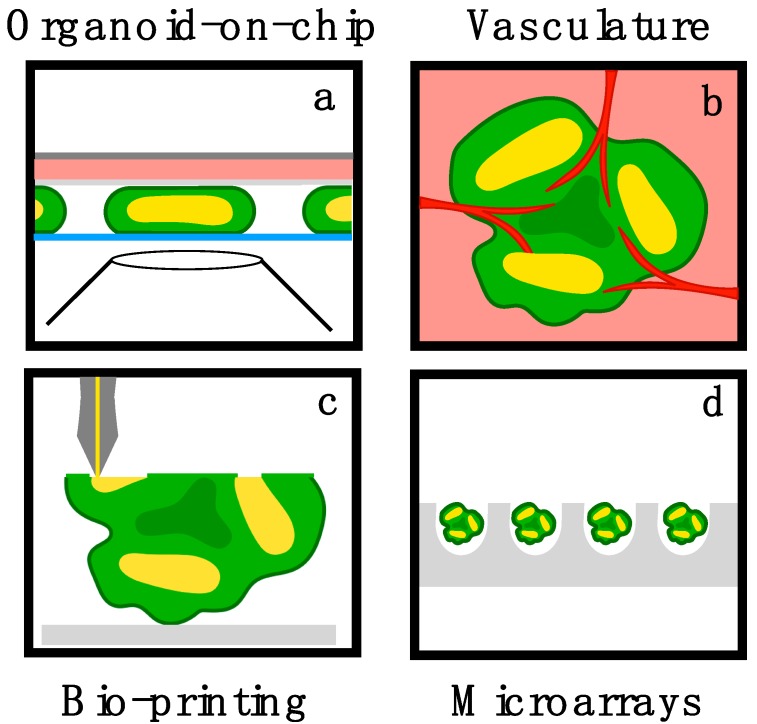
Bioengineering brain organoids. (**a**) On-chip approach to culture brain organoids in confined compartments for long-term live-imaging [7]. (**b**) Vasculature is essential to prevent necrosis in the organoid core, but have so far been attained only by transplanting organoids into mice brains [125]. (**c**) 3D printing and (**d**) microwell arrays are possible solutions to decrease variability between organoids cultures.

During embryonic development, complexity increases over time: the diversity of cell types rises, spatial organization becomes more composite, and the interactions with non-neuronal components becomes more intricate. Furthermore, embryonic development in humans occurs over many months. Thus, growing organoids to late developmental stages poses several experimental challenges together with engineering opportunities [122]. In this section we review several key challenges and discuss possible bioengineering solutions. One major challenge is the lack of vasculature in organoid systems. Organoids grow to a millimeter diameter within a month, and diffusion through the dense tissue is limited, leading to necrosis. So far, researchers have tackled this issue by generating flow in culture systems using tilting and rotary elements [11,14]. This approach results in reduced culture variability, and increased tissue growth. However, it does not directly solve the issue of nutrient transport through the organoid. Microfluidic devices have been previously designed for 3D spheroid cultures [123,124]. We have recently developed a microfabricated device to improve organoid culture conditions and enable long-term live-imaging (Figure 3a) [7,61]. The device consisted of a reaction chamber of 150μm height, confined between an imaging compatible coverslip and a semipermeable membrane, coupled to a media reservoir. The organoids were cultured in the reaction chamber, resulting in efficient nutrient diffusion in the confined vertical dimension, and unconstrained growth in lateral dimensions. The organoids successfully developed over weeks, revealing enrichment of cerebral cortex specific genes, and markers of neuronal differentiation. 

Efficient perfusion in a full 3D culture would require a synthetic vasculature scaffold [126,127]. This has been accomplished by embedding a sacrificial material layer in hydrogels [128,129,130], through 3D stamping [131], and via optical bioprinting [132]. A challenge in integrating these techniques with organoid protocols is that the cumbersome hydrogel scaffold may interfere with the self-organization of cells in the organoid. Another intriguing direction is the integration of brain organoids with biological vasculature (Figure 3b). This was recently achieved as brain organoids were transplanted in mouse brain, leading to successful vascularization of the organoid [125]. Formation of vasculature networks have been previously studied using microfluidic techniques [133] and three-dimensional bioprinting [134]. However, so far the in vitro integration of brain organoids and endothelial cells was limited [135]. 

A second challenge is the high variability between organoid samples and over different protocols. In animal models such as flies or mice, the developmental stages and timing are reproducible between individuals. However, when tissues are cultured outside of the context of the complete organ, the variation between samples is large and challenges our ability to perform quantitative studies. It is not at all obvious how to design a culture system, which on one hand will constrain organoid culture to yield reproducible dynamics, and on the other hand will preserve the self-organization capacity of the 3D culture. Experimental variability can arise from several sources: variations in biochemical media conditions, variations in the initial state of the sample, and spontaneous symmetry breaking of the system. Media composition is an important factor in organoid protocols, and the usage of undefined supplements such as serum and Matrigel can lead to increased variability. The supplement of patterning factors such as dual SMAD inhibition, reduces the heterogeneity of cell identifies. However we are still far from understanding the ‘optimal’ differentiation media composition, and how it should be changed over time. High-throughput screening of small molecules in combination with live imaging can greatly advance this issue. 

A bigger issue is the lack of defined initial experimental conditions. Typically, thousands of stem cells are aggregated into ‘embryoid bodies’. The exact number of cells, and the aggregation protocol, are typically poorly controlled. Efforts have been recently made to engineer the initial organoid size, shape and composition using microwell arrays, droplet-based microfluidics, 3D bioprinting, and chemically programmed tissue assembly (Figure 3c,d) [122,136,137,138]. Finally, the most interesting variability between samples seems to emerge due to spontaneous symmetry breaking during neural induction. During neural induction, numerous lumens spontaneously appear within the embryoid body [30,139]. The number of lumens in a single organoid varies from one to dozens, and may change over time. Each lumen forms a center of development, and the coexistence of multiple lumens in a single organoid forms a major departure from in vivo development. It seems that controlling lumen formation using small molecules or by reducing the initial sample size to the single cell limit will significantly reduce sample-to-sample variability. Here too, one can hope that advanced microfabrication and bioprinting techniques will offer a solution.

Finally, the embryonic brain receives multiple biochemical inputs and feedback from the rest of the body, which is missing in organoid cultures. Peripheral input and feedback are essential for development and network formation [140] and should be recapitulated in vitro. One possibility is the co-culture of organoids, such as neural tube organoids, with other tissues, such as muscle cells. The generation of functional skeletal muscle tissue from human pluripotent cell lines has been recently successfully implemented [141], and thus brain organoid and muscle tissue can be derived from the same cell line. Furthermore, neuromuscular junctions have been previously established in 2D cultures [142,143]. It would be interesting to see whether the formation of coupling between neuronal and muscular tissues, can significantly affect organoid development, by promoting neuronal maturation and activity.

## 5. Conclusions and Future Directions

Over the recent years we have witnessed major breakthroughs in stem cell technologies and genome editing, which enabled setting up models to study early human brain development in health and disease. Whereas there is an obvious trend to grow the organoids to longer periods, which allow for development of more mature neurons and a larger cell type repertoire, this usually comes with the complete lack of a defined structure. The challenges in this type of research are immense; our current information regarding human brain development is limited, therefore, the realization of how well the model fits with the real organ is somewhat lacking. We believe that further development of well-structured models will require multidisciplinary approaches. Combining of better understanding of how the human brain develops, what are the external and regional cues that induce development of a particular brain region, together with advanced bioengineering approaches may result in better models for specific brain regions.
